# Timing of Physical Activity and Cardiovascular Health: Opportunities and Challenges

**DOI:** 10.1016/j.jacadv.2024.101325

**Published:** 2024-10-07

**Authors:** Tongyu Ma

**Affiliations:** Department of Rehabilitation Sciences, The Hong Kong Polytechnic University, Hong Kong, China

**Keywords:** cardiovascular health, physical activity, time of day

**Figure:**
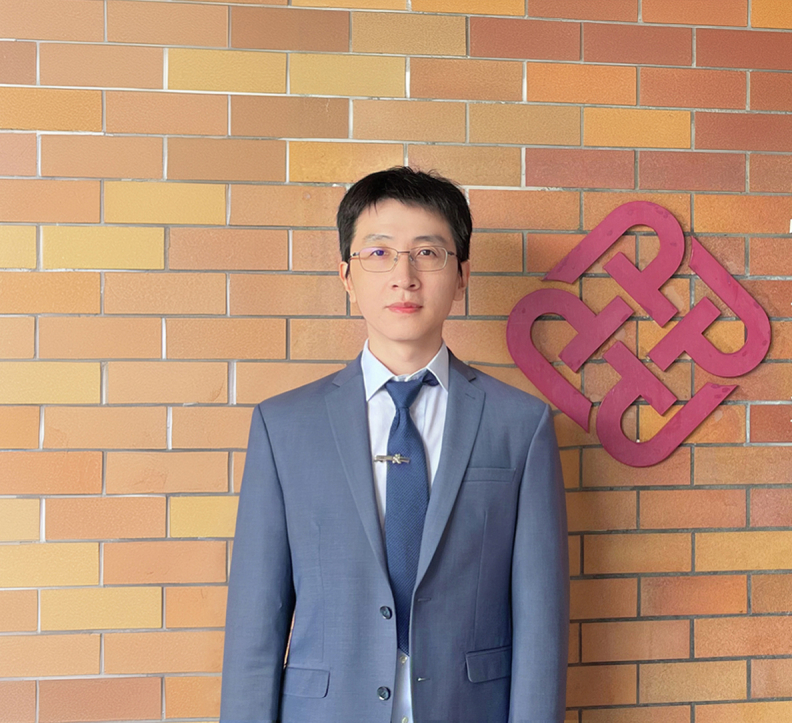

The global prevalence of sedentary lifestyles and inadequate physical activity (PA) poses a significant threat to health care systems and population well-being. Encouraging individuals to adopt healthy lifestyles and engage in sufficient PA remains a pressing task for clinicians, researchers, and stakeholders. Meeting the current guidelines, which recommend at least 150 minutes of moderate-intensity PA or 75 minutes of vigorous-intensity PA, or an equivalent combination of both, could have a profound impact on disease prevention, population longevity, and overall quality of life.^1^^,^^2^

The current PA guideline focuses primarily on the total amount of PA without fully considering the patterns of PA accumulation. This emphasis is rooted in the findings of epidemiological studies and short-term clinical trials, which have shown that PA volume, but not PA pattern, is the key predictor of health outcomes.^3^^,^^4^ However, a growing body of evidence suggests that the manner in which PA is accumulated is also crucially linked to health, presenting new opportunities for precision medicine and individualized PA prescriptions.^5^^,^^6^

A recent study conducted by Cominetti et al explored the relationship between PA timing and cardiovascular disease (CVD) risk through two distinct study designs.^7^ The cross-sectional component examined the association between PA timing and biomarkers, including blood pressure and lipids, while the cohort study investigated the relationship between PA timing and CVD incidence. The findings of this study revealed a 3-fold increase in CVD incidence among individuals whose PA peaked between 7 am and 12 am, compared to those whose PA peaked between 10 am and 2 pm, suggesting a potential hazard associated with morning PA compared to PA occurring later in the day.

It is essential to acknowledge that some PA is better than none, and this principle remains true even if PA occurs in the morning. This is particularly relevant for individuals who can only engage in PA during morning hours due to time constraints. However, the question of whether the morning, particularly the early morning,^6^ is an unfavorable time for exercise in terms of cardiovascular health presents a clinically important consideration. Physiological studies have reported a morning surge in blood pressure and augmented sympathetic nerve activity, which may exacerbate the PA-induced stress on the cardiovascular system, potentially leading to a temporary increased susceptibility to adverse CVD events.^8^^,^^9^

Cominetti's study also revealed that a PA pattern characterized by the absence of appreciable peaks over the waking period is associated with a three-fold increase in CVD risk compared to the cluster with PA peaking between 10 am and 2 pm. It is plausible that a PA pattern lacking peaks may be less health-enhancing because purposeful, structured PA, such as a 20-minute walk, a session of group exercise, or a game of tennis, typically leads to a peak in diurnal PA levels. The absence of peaks may suggest a lack of purposeful, structured PA. Notably, occupational PA is an important confounder in this outcome, as it has been shown to be nonhealth-enhancing and even detrimental.^10^ Given that occupational PA typically involves long-lasting, light-intensity activities, the cluster exhibiting no peak reported in Cominetti's study could be representing individuals whose PA were mainly accumulated in occupational settings.

Currently, the investigation of PA timing is still in its nascent stage, and several fundamental methodological challenges need to be addressed to enhance the quality of research in this field. One of the most pressing issues is the complexity of defining the timing of PA. A significant limitation of epidemiological studies in this area is the lack of an evidence-based approach to define PA timing. It is essential to conduct rigorous validation studies to standardize the algorithm for determining the time of day of PA.

Second, many studies rely on summary statistics from wearable devices, such as average acceleration, total counts, and total time spent on moderate-to-vigorous PA. However, these summary statistics provide limited insight into the specific human behaviors being examined. Recent advances in human movement recognition from accelerometer data offer a promising avenue for investigating the timing of PA. By leveraging artificial intelligence tools, such as machine learning and deep learning, it is possible to capture specific movements, including driving, walking, climbing stairs, and others.^11^ With the rapid technical advancements in accelerometer data processing, future studies may focus on examining the timing of specific activities, providing a more nuanced understanding of the relationship between PA and health.

Finally, the influence of PA timing may vary across different health outcomes. While morning PA has been suggested as an unfavorable time of day to engage in PA for CVD health, as the risk of CVD events tends to increase in those who engage in PA in the morning compared to those who are active in the mid-day, after accounting for total PA volume.^7^ In contrast, morning PA has appeared to be the optimal time of day to engage in PA for weight loss, potentially due to the lack of carbohydrate stores after overnight fasting, which may promote lipid oxidation during PA and facilitate weight loss.^12^ Future studies are warranted to investigate the optimal timing of PA for specific populations (eg, healthy participants vs individuals with pre-existing CVD) and various health outcomes (eg, CVD vs weight management) to provide tailored suggestions.

## Funding support and author disclosures

The author has reported that they have no relationships relevant to the contents of this paper to disclose.
